# Predatory Bacteria Attenuate *Klebsiella pneumoniae* Burden in Rat Lungs

**DOI:** 10.1128/mBio.01847-16

**Published:** 2016-11-08

**Authors:** Kenneth Shatzkes, Eric Singleton, Chi Tang, Michael Zuena, Sean Shukla, Shilpi Gupta, Sonal Dharani, Onoyom Onyile, Joseph Rinaggio, Nancy D. Connell, Daniel E. Kadouri

**Affiliations:** a Division of Infectious Disease, Department of Medicine, Rutgers New Jersey Medical School, Newark, New Jersey, USA; b Department of Oral Biology, Rutgers School of Dental Medicine, Newark, New Jersey, USA; c Department of Diagnostic Sciences, Rutgers School of Dental Medicine, Newark, New Jersey, USA

## Abstract

*Bdellovibrio bacteriovorus* and *Micavibrio aeruginosavorus* are predatory bacteria that naturally—and obligately—prey on other Gram-negative bacteria, and their use has been proposed as a potential new approach to control microbial infection. The ability of predatory bacteria to prey on Gram-negative human pathogens *in vitro* is well documented; however, the *in vivo* safety and efficacy of predatory bacteria have yet to be fully assessed. In this study, we examined whether predatory bacteria can reduce bacterial burden in the lungs in an *in vivo* mammalian system. Initial safety studies were performed by intranasal inoculation of rats with predatory bacteria. No adverse effects or lung pathology were observed in rats exposed to high concentrations of predatory bacteria at up to 10 days postinoculation. Enzyme-linked immunosorbent assay (ELISA) of the immune response revealed a slight increase in inflammatory cytokine levels at 1 h postinoculation that was not sustained by 48 h. Additionally, dissemination experiments showed that predators were efficiently cleared from the host by 10 days postinoculation. To measure the ability of predatory bacteria to reduce microbial burden *in vivo*, we introduced sublethal concentrations of *Klebsiella pneumoniae* into the lungs of rats via intranasal inoculation and followed with multiple doses of predatory bacteria over 24 h. Predatory bacteria were able to reduce *K. pneumoniae* bacterial burden, on average, by more than 3.0 log_10_ in the lungs of most rats as measured by CFU plating. The work presented here provides further support for the idea of developing predatory bacteria as a novel biocontrol agent.

## IMPORTANCE

A widely held notion is that antibiotics are the greatest medical advance of the last 50 years. However, the rise of multidrug-resistant (MDR) bacterial infections has become a global health crisis over the last decade. As we enter the postantibiotic era, it is crucial that we begin to develop new strategies to combat bacterial infection. Here, we report one such new approach: the use of predatory bacteria (*Bdellovibrio bacteriovorus* and *Micavibrio aeruginosavorus*) that naturally—and obligately—prey on other Gram-negative bacteria. To our knowledge, this is the first study that demonstrated the ability of predatory bacteria to attenuate the bacterial burden of a key human pathogen in an *in vivo* mammalian system. As the prevalence of MDR infections continues to rise each year, our results may represent a shift in how we approach treating microbial infections in the future.

## INTRODUCTION

The identification of penicillin by Alexander Fleming in 1928 ushered in the golden age of antibacterial treatment and is widely regarded as the greatest medical advance of the last 50 years (1). However, due to the overuse of our antibiotic supplies, among other reasons, the rise of multidrug-resistant (MDR) bacterial infections has become a global health crisis over the last decade (2). The issue of MDR infections and the lack of antibiotics in the development pipeline have spurred researchers to consider new ways to combat bacterial infection in the coming postantibiotic era. One such approach is the use of naturally occurring predatory bacteria.

*Bdellovibrio bacteriovorus* and *Micavibrio aeruginosavorus* are small Gram-negative proteobacteria that are obligate predators of other Gram-negative bacteria (3, 4). *B. bacteriovorus* randomly moves through the environment via the use of a single, polar flagellum and invades across the prey outer cell membrane into the periplasmic space of the Gram-negative prey, establishing a bdelloplast. The mechanism by which *B. bacteriovorus* bacteria invade their prey is not completely elucidated, but type IV pili have been implicated as essential in the process (5–7). Once inside, *B. bacteriovorus* grows in a filamentous manner by digesting the prey cell from within, divides into a number of progeny, and then lyses the bdelloplast to continue looking for more prey to invade. *M. aeruginosavorus* are epibiotic predators that do not invade their prey. Instead, they attach themselves to the outer membrane of a prey cell and digest the contents in a “vampire”-like fashion (4, 8, 9).

Multiple studies have already demonstrated the effectiveness with which both *B. bacteriovorus* and *M. aeruginosavorus* are able to control key human pathogens, including in MDR infections (10), *in vitro* (11–14). Predatory bacteria are unable to invade and prey on mammalian cells (15), mitigating potentially harmful off-target effects. Additionally, predatory bacteria are nonpathogenic in a variety of animal models, including mice, rabbits, guinea pigs, and chicks (14, 16–19). Furthermore, development of genetically stable resistance to predation has yet to be confirmed (20). However, most studies examining the ability of predatory bacteria to control human pathogens have been performed *in vitro*; thus, the efficacy with which predatory bacteria can control a bacterial infection in a live host is still not known.

In this study, first, the safety of intranasal inoculation of predatory bacteria in Sprague-Dawley (SD) rats was assessed. Then, lungs of rats were exposed to *Klebsiella pneumoniae* and were treated with multiple doses of predatory bacteria to determine their ability to reduce bacterial burden within the lungs. To our knowledge, this is the first study that demonstrated the ability of predatory bacteria to attenuate the bacterial burden of a key human pathogen in an *in vivo* mammalian system. The work presented here further supports the potential development of predatory bacteria into a biocontrol agent.

## RESULTS

### Host morbidity.

While the safety of administering predatory bacteria into the lungs of mice has already been demonstrated (19), we began by investigating if the predators are compatible with the animal model being used in this study, Sprague-Dawley (SD) rats. To examine the effect on host morbidity of introducing predatory bacteria into the respiratory tract of SD rats, we performed intranasal inoculations of 6.0 × 10^8^ PFU/rat of *B. bacteriovorus* 109J, 1.1 × 10^9^ PFU/rat of *B. bacteriovorus* HD100, or 5.0 × 10^7^ PFU/rat of *M. aeruginosavorus* ARL-13 into rats in three groups containing six rats each. Another group of six rats was inoculated as a control with the vehicle, phosphate-buffered saline (PBS).

The animals were housed and monitored for up to 10 days postinoculation for any signs of discomfort, illness, or infection. At 10 days, all 18 rats inoculated with PBS or either strain of *B. bacteriovorus* were visually healthy with no apparent signs of illness (Table 1). One rat inoculated with *M. aeruginosavorus* died immediately after inoculation; however, veterinary consultation proposed asphyxiation as the probable cause of death. Blood analysis of the specific rat confirmed no elevated concentrations of white and red blood cell counts, and red blood cells were found with normal morphology (data not shown), suggesting that death was not caused by an acute infection resulting from *M. aeruginosavorus* inoculation. The remaining five rats inoculated with *M. aeruginosavorus* were found to be healthy at 10 days postinoculation (Table 1).

**TABLE 1:** Numbers of rats showing visual signs of morbidity after intranasal inoculation with predatory bacteria

Treatment	No. of rats showing visual signs of morbidity at indicated time after inoculation/total no. of rats			
	1 h	24 h	48 h	10 days
Control (PBS)	0/12	0/12	0/12	0/6
*B. bacteriovorus* 109J	0/12	0/12	0/12	0/6
*B. bacteriovorus* HD100	0/12	0/12	0/12	0/6
*M. aeruginosavorus* ARL-13	0/12	0/12	0/12	1/6^a^
*K. pneumoniae^b^*	0/8	0/8	0/8	

a One rat died immediately after inoculation with predatory bacteria; however, asphyxiation was suggested as the cause of death.

b With animal well-being in mind, rats inoculated with *K. pneumoniae* were not kept past 48 h postinoculation.

**FIG 1:**
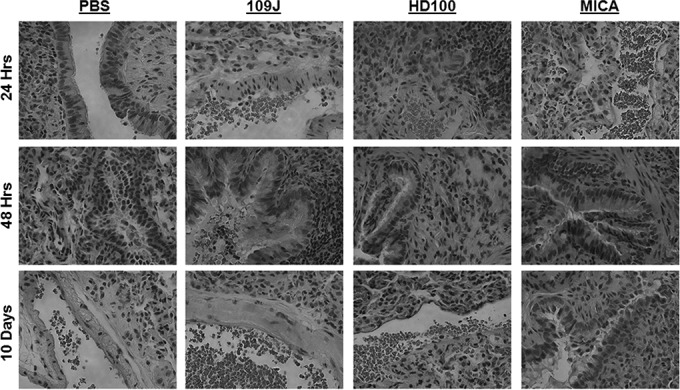
Histological examination of rat lungs after intranasal introduction of predatory bacteria. *B. bacteriovorus* 109J or HD100 bacteria or *M. aeruginosavorus* ARL-13 (MICA) bacteria were introduced into the lungs of SD rats via intranasal inoculation. Histological examination of rat lungs revealed no pathological abnormalities compared to rats inoculated with the control (PBS). All images are representative micrographs taken at 24, 48 h, and 10 days postintranasal inoculation and at ×40 total magnification.

Histological examination of lung tissue from the 10-day exposure experiment revealed no pathology due to inoculation with predatory bacteria in any treatment group (Fig. 1). All specimens were characterized by lungs showing partial collapse of alveolar sacs without signs of inflammation or other abnormalities. Together, the data suggest that when inhaled, predatory bacteria have no adverse effects on rat morbidity.

### Inflammatory response and histopathology.

To examine the effect of predatory bacteria on the host immune response within the lungs of SD rats, we introduced PBS, 3.8 × 10^8^ PFU/rat of *B. bacteriovorus* 109J, 2.5 × 10^8^ PFU/rat of *B. bacteriovorus* HD100, or 2.0 × 10^8^ PFU/rat of *M. aeruginosavorus* ARL-13 into four groups of 36 rats each. An additional 28 rats were inoculated with a sublethal dose of 2.8 × 10^7^ CFU/rat of *K. pneumoniae*, a known respiratory pathogen. Animals were again visually monitored for any signs of illness for up to 48 h. All 108 animals inoculated with any strain of predatory bacteria were found to be healthy for the duration of the experiment (Table 1). Twelve animals from each group (eight for the *K. pneumoniae* group) were sacrificed at 1, 24, and 48 h postinoculation, and the lung samples were harvested for histological examination and to assess inflammatory cytokine levels.

All rats that were inoculated with predatory bacteria did not show any visual signs of illness or discomfort. Histological examination of lung tissue revealed no abnormal pathology due to *B. bacteriovorus* 109J or *M. aeruginosavorus* at 24 h postinoculation (Fig. 1). At 48 h, examined lungs from rats inoculated with *B. bacteriovorus* 109J or *M. aeruginosavorus* showed only mild acute inflammation with stromal eosinophil infiltration and bronchioles containing a greater than normal amount of a proteinaceous substance consistent with mucus (Fig. 1). Lungs inoculated with *B. bacteriovorus* HD100 exhibited reactive lymphoid hyperplasia and acute inflammation at 24 h postinoculation; however, no pathological abnormalities were seen at 48 h (Fig. 1). Lungs inoculated with *K. pneumoniae* showed no abnormalities at 24 h postinfection; in contrast, at 48 h, lungs exhibited acute inflammation, stromal infiltration of eosinophils, germinal center formation within the lymphoid component of the inflammatory infiltrate, and bronchioles with a greater than normal amount of proteinaceous substance consistent with mucus. Lungs inoculated with the vehicle, PBS, exhibited no histological abnormalities at any time point examined.

**FIG 2:**
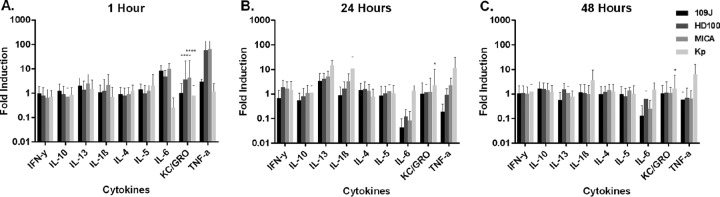
Inflammatory protein profile within rat lungs in response to intranasal inoculation of predatory bacteria. ELISA analysis of responses of IL-1β, IL-4, IL-5, IL-8, IL-10, IL-13, CXCL-1/KC, gamma interferon (IFN-γ), and TNF to intranasal inoculation of predatory bacteria relative to those seen with a PBS control was performed. *B. bacteriovorus* 109J or HD100 bacteria or *M. aeruginosavorus* ARL-13 bacteria (and also *K. pneumoniae* [Kp] bacteria as a control) were introduced into the lungs of SD rats via intranasal inoculation. Inflammatory proteins were assessed within the lungs at (A) 1, (B) 24, and (C) 48 h postinoculation. Twelve rats per treatment group (eight for *K. pneumoniae*) were used at each time point. Data are combined from two independent experiments. Data represent means ± standard errors of the means. Significant differences between treatment groups and respective control were determined using ANOVA (*, *P* < 0.05; ****, *P* < 0.0001).

Enzyme-linked immunosorbent assay (ELISA) analysis of inflammatory proteins revealed 59.3- and 63.9-fold increases of tumor necrosis factor alpha (TNF-α) levels and 3.7- and 4.4-fold increases of KC/GRO (CXCL1) levels at 1 h postinoculation in rats inoculated with *B. bacteriovorus* HD100 and *M. aeruginosavorus*, respectively (Fig. 2A). However, none of the increases were sustained, as the levels of TNF-α and KC/GRO returned to baseline by 24 h postinoculation (Fig. 2B). We also observed 8.6-, 4.8-, and 10.0-fold increases in levels of interleukin-6 (IL-6) in rats inoculated with *B. bacteriovorus* 109J, *B. bacteriovorus* HD100, and *M. aeruginosavorus*, respectively, at 1 h postinoculation (Fig. 2A), but again, levels returned to baseline by 24 h (Fig. 2B). IL-13 levels increased 3.5-, 4.1-, and 5.2-fold in rats treated with *B. bacteriovorus* 109J, *B. bacteriovorus* HD100, and *M. aeruginosavorus*, respectively, at 24 h postinoculation (Fig. 2B) before reverting to physiological levels at 48 h (Fig. 2C). Rats inoculated with *M. aeruginosavorus* also demonstrated a 3.3-fold increase in IL-1β levels at 24 h (Fig. 2B). No levels of other inflammatory cytokines were found to be increased more than 3.0-fold at any time point examined in any group treated with predatory bacteria (Fig. 2). In contrast, rats inoculated with *K. pneumoniae* still exhibited 14.4-, 11.0-, and 11.4-fold increases in the levels of IL-13, IL-1β, and TNF-α, respectively, at 24 h postinoculation (Fig. 2B). At 48 h postinoculation, levels of IL-1β and TNF-α were still found to be substantially increased (3.6- and 6.3-fold, respectively) (Fig. 2C). Collectively, the data indicate that administering predatory bacteria at high concentrations into the lungs of rats does not result in adverse tissue pathology or provoke a sustained inflammatory response.

### Predatory bacterial dissemination.

To determine the predatory bacterial load within the lungs and other organs at 1, 24, and 48 h and 10 days post-intranasal inoculation, lung, liver, kidney, and spleen samples were also harvested during the previously described experiments and analyzed for the presence of predatory bacteria 16S rRNA using quantitative PCR (qPCR). Rats sacrificed at 1, 24, or 48 h were inoculated as described for the inflammatory response and histopathology experiment, while rats sacrificed at 10 days were treated as described above (see “Host morbidity”).

**FIG 3:**
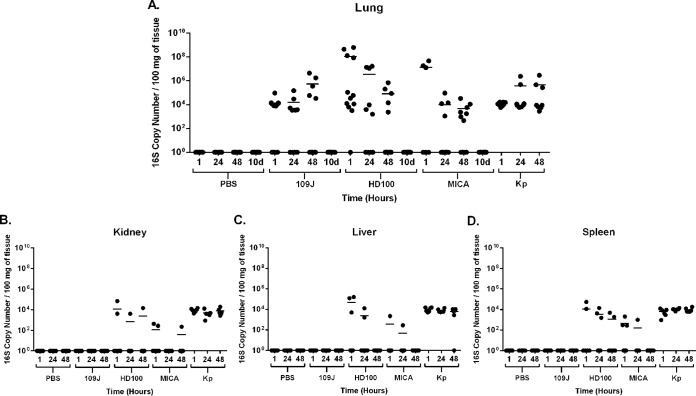
Predatory bacterial dissemination within hosts. qPCR detection of predatory bacteria within the host was performed. The (A) lungs, (B) kidneys, (C) livers, and (D) spleens were probed for *B. bacteriovorus* 109J and HD100, *M. aeruginosavorus* (MICA), and *K. pneumoniae* at 1, 24, and 48 h and 10 days (d) (lung only) postinoculation. Twelve rats per treatment group (eight for *K. pneumoniae*) were analyzed at each time point for the lungs, with the exception of the *M. aeruginosavorus* 1-h treatment group, which had six rats. Only six rats per treatment group were analyzed for the kidney, liver, and spleen. Each data point represents a single rat’s respective bacterial load. A line represents the mean of the results from each treatment set. Data are combined from the results of two independent experiments.

At 1 h postinoculation, *B. bacteriovorus* 109J was detected in the lungs of 7/12 rats (at levels ranging from 7.5 × 10^3^ to 9.2 × 10^4^ copy numbers), *B. bacteriovorus* HD100 in 11/12 rats (3.1 × 10^3^ to 6.1 × 10^8^), and *M. aeruginosavorus* in 3/6 rats (1.3 × 10^7^ to 4.6 × 10^7^) (Fig. 3A). Samples were available for only 6 (of 12) of the rats inoculated with *M. aeruginosavorus* at the 1 h time point. Detectable levels of *B. bacteriovorus* HD100 and *M. aeruginosavorus* in animals decreased with time. At 48 h, *B. bacteriovorus* HD100 was detected in only 5/12 rats (at levels ranging from 2.4 × 10^3^ to 2.0 × 10^5^ copy numbers) and *M. aeruginosavorus* in 7/12 rats (4.6 × 10^2^ to 3.2 × 10^4^) (Fig. 3A). By 10 days postinoculation, no *B. bacteriovorus* HD100 or *M. aeruginosavorus* bacteria were detected in any rats inoculated (Fig. 3A). Interestingly, while *B. bacteriovorus* 109J was detected in the lungs of only 5/12 rats at 48 h postinoculation, the average level of *B. bacteriovorus* 109J actually increased to 5.4 × 10^5^ copy numbers (Fig. 3A). However, this increase was not sustained, as no *B. bacteriovorus* 109J was detected in the lungs of rats at 10 days postinoculation (Fig. 3A). In comparison, *K. pneumoniae* 16S rRNA was detected in the lungs of all rats at every time point examined and at higher average levels (1.2 × 10^4^ to 4.6 × 10^5^ copy numbers; Fig. 3A).

Predatory bacteria 16S rRNA was detected in only a limited number of the kidneys, livers, and spleens of rats inoculated with either *B. bacteriovorus* HD100 or *M. aeruginosavorus* at 1 h postinoculation (Fig. 3B to D). The kidneys, livers, and spleens of only six rats per treatment group at each time point were assessed. The trend exhibited a decrease in the concentration of predatory bacteria, as well as a decrease in the number of animals with predators that disseminated from the lungs to other organs examined. By 48 h postinoculation, *B. bacteriovorus* HD100 was detected in the kidneys of only 1/6 rats, in the livers of 0/6 rats, and the kidneys of only 2/6 rats (Fig. 3B to D). Similarly, *M. aeruginosavorus* was detected in the kidneys of only 1/6 rats and in the livers and kidneys of none of the rats inoculated at 48 h (Fig. 3B to D). No *B. bacteriovorus* 109J was detected in the kidneys, liver, or spleen of any rat inoculated at any time point assessed (Fig. 3B to D). In contrast, *K. pneumoniae* disseminated at high levels to the kidney, liver, and spleen in all rats inoculated by 1 h postinfection and continued to be detectable at similarly high levels at 48 h (Fig. 3B to D). In conclusion, the data suggest that predatory bacterial dissemination to other organs after respiratory inoculation is limited, while predators are quickly and efficiently cleared from the host by 10 days postinoculation.

### Pathogen inoculation and treatment.

To determine whether predatory bacteria are able to reduce bacterial burden in the lungs, 36 rats were introduced with 3.3 × 10^7^ CFU/rat of *K. pneumoniae* via intranasal inoculation (“experimental group”). Thirty-six more rats were inoculated with PBS (“control group”). Twelve rats each from the control and experimental groups were dosed four times with PBS, 4.6 × 10^8^ PFU/rat of *B. bacteriovorus* 109J, or 6.6 × 10^7^ PFU/rat of *M. aeruginosavorus* ARL-13 over a 24-h period postinfection. Dosages of PBS or predatory bacteria were administered through intranasal inoculation at 30 min and 6, 12, and 18 h before animals were sacrificed and organs harvested at 24 h postinoculation.

**FIG 4:**
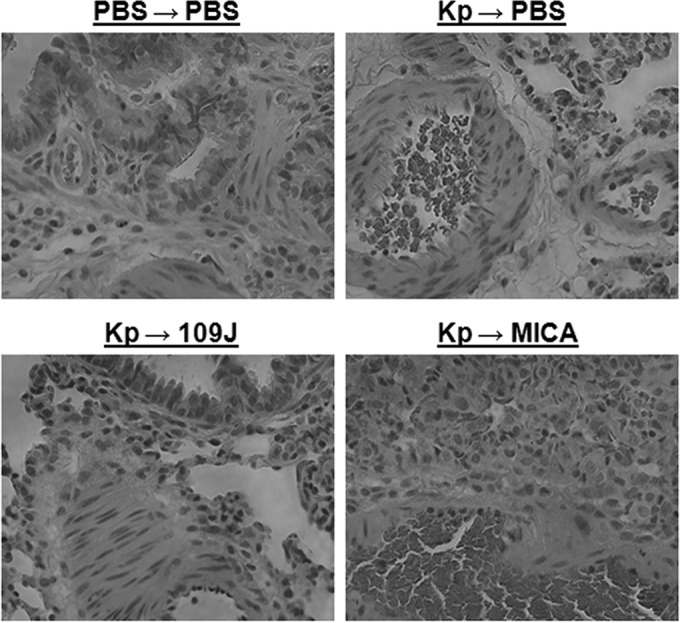
Histological examination of rat lungs after treatment of *K. pneumoniae* inoculation with predatory bacteria. *K. pneumoniae* (or PBS for control groups) was initially introduced into the lungs of rats via intranasal inoculation. Animals were then treated with PBS, *B. bacteriovorus* 109J, or *M. aeruginosavorus* (MICA) at 30 min and 6, 12, and 18 h postinoculation. All images are representative micrographs taken at 24 h postinoculation and at ×40 total magnification.

As before, examination of lung tissue revealed no significant histological abnormalities due to predatory bacteria at 24 h postinoculation. Tissue from rats inoculated with *K. pneumoniae* and treated with either *B. bacteriovorus* 109J or *M. aeruginosavorus* was similar to the tissue from the PBS-inoculated control group and generally showed peribronchiolar aggregates of eosinophils and macrophages, though a few samples showed partial collapse of the alveolar sacs (Fig. 4). Rats inoculated with *K. pneumoniae* and not treated with predatory bacteria exhibited bronchiole-associated lymphoid tissue with reactive lymphoid hyperplasia, as well as peribronchiolar infiltration of eosinophils (Fig. 4).

**FIG 5:**
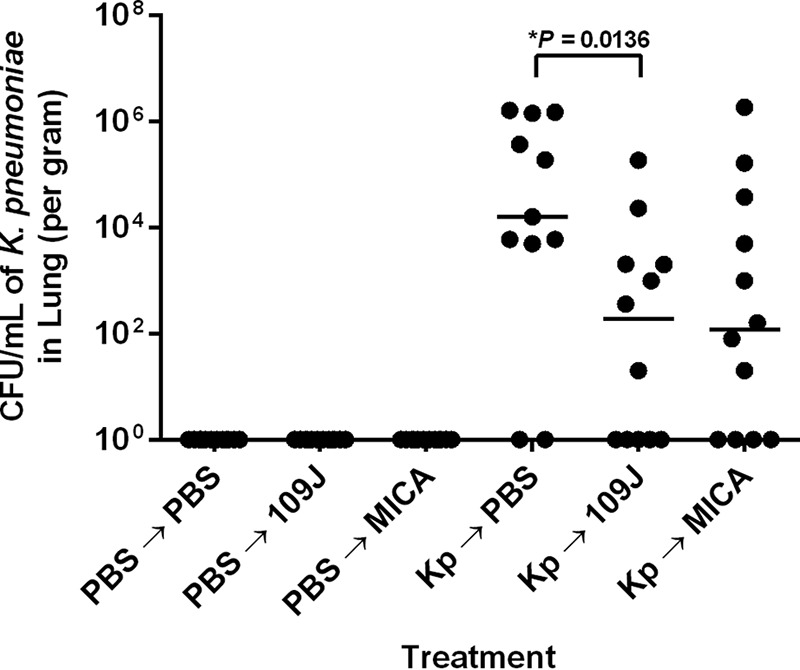
*K. pneumoniae* bacterial burden within lungs of rats after treatment with predatory bacteria. *K. pneumoniae* (or PBS for control groups) was initially introduced into the lungs of rats via intranasal inoculation. Animals were then treated with PBS, *B. bacteriovorus* 109J, or *M. aeruginosavorus* (MICA) at 30 min and 6, 12, and 18 h postinoculation. At 24 h, lungs were harvested, homogenized, and plated on MacConkey agar plates to recover *K. pneumoniae* CFU. Twelve rats per treatment group were used at each time point. Each data point represents a single rat’s respective bacterial load. Horizontal lines represent the median of the results from each treatment set. Data are combined from the results from two independent experiments. Analyses of significant differences between treatment groups and respective controls were performed using the Mann-Whitney test (*, *P* < 0.05).

Lung samples were homogenized and plated on MacConkey agar. As expected, no colonies of *K. pneumoniae* from the lungs of rats in the PBS-inoculated control group were isolated on agar plates (Fig. 5). In the experimental group, a median of 1.6 × 10^4^ CFU/ml (mean = 4.7 × 10^5^ CFU/ml) of *K. pneumoniae* was isolated from the lungs of 75.0% of rats initially inoculated with *K. pneumoniae* and treated with PBS (Fig. 5). In contrast, we recovered colonies of *K. pneumoniae* from the lungs of only 58.3% of rats inoculated with *K. pneumoniae* and treated with *B. bacteriovorus* 109J, with a median of 1.9 × 10^2^ CFU/ml (mean = 1.8 × 10^4^ CFU/ml) (Fig. 5). *K. pneumoniae* was also recovered from the lungs of 66.6% of rats treated with *M. aeruginosavorus*, with a median of 1.2 × 10^2^ CFU/ml (mean = 1.7 × 10^5^ CFU/ml). However, for both the *B. bacteriovorus* 109J and *M. aeruginosavorus* treatments, levels of *K. pneumoniae* CFU showed high variability. Strikingly, 83.3% of *B. bacteriovorus* 109J-treated rats and 66.6% of *M. aeruginosavorus*-treated rats exhibited greater than 3.0 log_10_ reductions in copy numbers of *K. pneumoniae* recovered compared to the mean of the results from PBS-treated rats (Fig. 5). Furthermore, we recovered no *K. pneumoniae* from 41.6% of *B. bacteriovorus* 109J-treated rats and 33.3% of *M. aeruginosavorus*-treated rats. Taken together, the data indicate that *B. bacteriovorus* 109J and *M. aeruginosavorus* are able to efficiently reduce bacterial burden in the lungs of rats.

## DISCUSSION

The prevalence of antibiotic-resistant infections has climbed to frightening levels over the last decade (2). Compounding this problem is the fact that since the development of linezolid in the 1970s, virtually no new class of antibiotics, particularly those that can target Gram-negative bacterial pathogens, has been discovered through traditional drug screening techniques. Recently, the first MDR infection caused by a member of the *Enterobacteriaceae* harboring the *mcr-1* plasmid-borne colistin resistance gene was detected in the United States, potentially signaling the emergence of truly pan-drug-resistant bacteria (21). For these reasons, among others, it is crucial that we begin to develop new treatments to combat bacterial infection. Researchers have already begun investigating potential new antimicrobial strategies (22), such as the use of antimicrobial peptides (23, 24), phage therapy (25, 26), and gene-editing enzymes (27, 28). Here, we report another promising novel approach: the use of predatory bacteria to control Gram-negative human pathogens.

In this study, we first determined the safety of intranasal inoculation of predatory bacteria in rats. High doses (>10^7^ PFU/ml) of *B. bacteriovorus* 109J or HD100 or of *M. aeruginosavorus* ARL-13 were administered via intranasal inoculation into the lungs of SD rats. Two different *B. bacteriovorus* strains were examined to ensure that the results were not strain specific; unfortunately, we were unable to obtain additional *M. aeruginosavorus* strains. Of the total of 126 rats inoculated with predatory bacteria during the safety experiments, 125 rats were healthy, with no visual signs of adverse effects. Histological examination of tissue also revealed no adverse pathology associated with respiratory inoculation of predatory bacteria.

The lack of toxicity due to inoculation with predatory bacteria is in agreement with previous studies demonstrating the safety of introducing predatory bacteria in a variety of animal models (16–19). In particular, we observed similar results in our previous study, in which C57BL/6 mice were intranasally inoculated with high concentrations of viable or heat-killed *B. bacteriovorus* or *M. aeruginosavorus* (19). In that study, no mouse exhibited either morbidity or any histological abnormalities at up to 50 days postinoculation, signaling that predatory bacteria are nontoxic when inhaled.

We next looked to determine the immune response to predatory bacteria within the lungs of rats. A slight increase in levels of inflammatory cytokines (IL-1β, IL-6, IL-13, TNF-α, KC/GRO) after inoculation with predatory bacteria was observed at the earlier time points of 1 and 24 h; however, inflammatory protein levels returned to baseline levels by 48 h postinoculation. The cytokine response in rats inoculated with the positive control, *K. pneumoniae*, was not as highly elevated as the response reported in other studies (29, 30). An explanation for this is the fact we used a sublethal dose to ensure that the animals would not succumb to the infection before administration of predatory bacteria in the lung infection model. Furthermore, we demonstrated that predatory bacteria do not disseminate efficiently to other organs and are quickly cleared from the lungs of the host by 10 days postinoculation. As the aforementioned inflammatory cytokines are key players in the primary immune response, we suspect that the predatory bacteria are promptly and efficiently cleared through innate immunity mechanisms.

Our results seen with the rat model align with our previous study, where intranasal or intravenous inoculation of high doses of either *B. bacteriovorus* or *M. aeruginosavorus* in mice caused no tissue pathology and induced only a modest inflammatory response that returned to baseline levels within 24 h postinoculation (19). Furthermore, predatory bacteria were completely cleared from the animals by an innate immune response (possibly via neutrophils) within 48 h postinoculation (19). The consistency in our results provides further evidence that *B. bacteriovorus* and *M. aeruginosavorus* are inherently nonpathogenic in mammalian models. The lack of a productive and sustained immune response to predatory bacteria may be partially a result of the presence of an altered lipopolysaccharide (LPS) (31). The negatively charged phosphate residues of the LPS on the surface of Gram-negative bacteria are primarily responsible for antagonizing an immune response by strongly binding to Toll-like receptors on immune cells. *B. bacteriovorus* HD100 is known to express a neutrally charged LPS which has been shown to provoke only a weak inflammatory response *in vitro* (31), which may explain the lack of immunogenicity in our *in vivo* models. It is still unknown if *M. aeruginosavorus* expresses an altered LPS, as well.

The main objective of our study was to determine whether predatory bacteria are able to reduce the bacterial burden in the lungs in an *in vivo* mammalian system. We intranasally inoculated rats with *K. pneumoniae* and treated them with PBS, *B. bacteriovorus* 109J, or *M. aeruginosavorus* at 30 min and 6, 12, and 18 h postinoculation. In order to limit the number of animals being sacrificed, only *B. bacteriovorus* 109J was used in efficacy experiments. It was predicted that *B. bacteriovorus* 109J would provide a greater chance of reducing the bacterial burden than strain HD100 due to its weak innate immune response profile and its ability to remain longer in the lungs of the rats, as measured in dissemination experiments. Histological examination of lung tissue exhibited no significant pathological abnormalities due to treatment with predatory bacteria at 24 h postinoculation. Lung samples were homogenized and plated on MacConkey agar, a selective medium used to isolate Gram-negative and enteric bacteria, in order to determine *K. pneumoniae* concentrations (32). While the average reduction of *K. pneumoniae* bacterial burden due to treatment with *B. bacteriovorus* 109J or *M. aeruginosavorus* was approximately ≤1 log_10_, 83.3% of *B. bacteriovorus* 109J-treated rats and 66.6% of *M. aeruginosavorus*-treated rats exhibited greater than 3.0 log_10_ reductions in the levels of *K. pneumoniae* recovered compared to the mean of the results seen with PBS-treated rats. Furthermore, 5/12 *B. bacteriovorus* 109J-treated rats and 4/12 of *M. aeruginosavorus*-treated rats had no detectable *K. pneumoniae*, suggesting clearance of the pathogen from the host. The observed amount of *K. pneumoniae* reduced by predatory bacteria *in vivo* is similar to the reported change *in vitro*. One such study demonstrated the ability of predatory bacteria to reduce levels of five different strains of *K. pneumoniae* (10). *B. bacteriovorus* 109J and *M. aeruginosavorus* were able to reduce *K. pneumoniae* levels *in vitro* by averages of 3.4 and 3.0 log_10_ CFU/ml, respectively (10). Although active predation could be the sole contributor to the reduction of the *K. pneumoniae* load in the animals treated with predatory bacteria, one might suggest that additional immune response elements, elicited by the presence of the predatory bacteria, also played a role in reducing the microbial burden. Thus, the potential synergistic effects of predatory bacteria and the immune system should be a basis of future studies.

We are aware of only one other study that has tested the *in vivo* efficacy of treatments using predatory bacteria. That previous study determined whether oral administration of *B. bacteriovorus* could reduce the level of colonizing *Salmonella* in the guts of young chicks (18). The authors observed an average of 0.64 to 1.09 log_10_ reduction of levels of *Salmonella* (18). While the mean reductions in the chick study were similar to the mean values that we obtained, a major difference in our study was that the majority of rats treated with predatory bacteria showed a greater than 3.0 log_10_ reduction in *K. pneumoniae* levels. Note that, in the previous study, chicks were dosed once with predatory bacteria, whereas we dosed the rats four times over 24 h, modeling the dosing regimen in a typical antibiotic dosing schedule. Another major difference between the studies was in the physiological conditions of the animals, as birds have a physiological body temperature of 42°C. Predatory bacteria may prey upon the host bacteria and grow more efficiently in a mammal whose physiological temperature is 37°C, which is closer to the optimum predation conditions for predatory bacteria at 30°C. Furthermore, it is difficult for predatory bacteria to survive passage through the acidic environment of the stomach, and while an antacid was administered concurrently with predatory bacteria to increase the pH in the chick study, it is possible that the overall conditions of the chick gut are not optimal for predation.

It is important to emphasize the limitations of our model for extrapolation to the treatment of humans. We acknowledge that treatment with predatory bacteria at 30 min post-*K. pneumoniae* inoculation does not represent abrogation of an established infection. Rather, this study was an experimental exercise designed to determine whether predatory bacteria have the ability to reduce bacterial burden in an *in vivo* mammalian system. Our treatment scheme is similar to that of a recent study examining the efficacy of structurally nanoengineered antimicrobial peptide polymers (SNAPPs) in reducing MDR infections in mice (33). Mice were “infected” with a pathogen (*Acinetobacter baumannii*) and, similarly to our study method, were treated with SNAPPs at 30 min and 4 and 8 h postinoculation before being euthanized at 24 h.

In conclusion, our results indicate that predatory bacteria are safe to administer intranasally to a mammalian host, are able to attenuate pathogen burden in the lungs of rats, and may provide a novel way to combat infection caused by Gram-negative pathogens. Future studies will focus on using established *in vivo* models of infection to further determine whether the use of predatory bacteria is a viable treatment for Gram-negative infections, including MDR infections.

## MATERIALS AND METHODS

### Bacterial strains and growth conditions.

Predatory bacteria examined in this study were *Bdellovibrio bacteriovorus* strain 109J (ATCC 43826), *Bdellovibrio bacteriovorus* strain HD100 (ATCC 15356) (34), and *Micavibrio aeruginosavorus* strain ARL-13 (9). As the pathogen, *Klebsiella pneumoniae* ATCC 43816 was used and grown in Luria-Bertani (LB) medium (35). Predatory bacteria were cultured and processed as previously described (11, 15). *Escherichia coli* WM3064, a diaminipimelic acid (DAP) auxotroph, was used as prey and grown overnight in LB medium supplemented with 0.3 mM DAP. Predator stock lysates were made by coculturing the predators with prey cells in HEPES buffer (25 mM) supplemented with 3 mM MgCl_2_ and 2 mM CaCl_2_. Cocultures were incubated at 30°C until the culture cleared (stock lysates). In order to obtain high concentrations of *B. bacteriovorus* for inoculation experiments, 10 ml of a washed overnight culture of *E. coli* WM3064 cells (~1 × 10^9^ CFU/ml) was resuspended in 80 ml of HEPES medium containing 10 ml of predatory bacteria from the stock lysates and incubated for 24 h on a rotary shaker. To obtain higher *M. aeruginosavorus* concentrations, *M. aeruginosavorus* cocultures were prepared in 200 ml of HEPES medium containing 25 ml of prey and 25 ml of *M. aeruginosavorus* stock lysates and incubated on a rotary shaker for 72 h. Cocultures were passed two times through a 0.45-µm-pore-size Millex filter (Millipore) to remove residual prey and cell debris (filtered lysate). Filtered lysates were pelleted three times by centrifugation at 29,000 × *g* for 45 min using a Sorvall LYNX 4000 centrifuge (Thermo Fisher Scientific Inc.) to further purify and concentrate predator samples. Each time, the pellet was washed and resuspended in 50 ml of phosphate-buffered saline (PBS). For the final wash, the predator pellet was resuspended in 1 to 2 ml of PBS solution to reach final optical densities at 600 nm (OD_600_) of 0.2 ± 0.02 for *B. bacteriovorus* and 0.1 ± 0.02 for *M. aeruginosavorus*, which corresponded to PFU values of between ~5 × 10^9^ and 5 × 10^10^ PFU/ml and between ~5 × 10^8^ and 5 × 10^9^ PFU/ml, respectively. The standard double-layered agar method was used to determine predator cell concentrations (36). Fifty microliters of the predator samples was plated on DAP-supplemented LB agar and tryptic soy broth (TSB)-blood plates to confirm that the samples were free of prey cells and contaminants. Since the predatory bacteria were used directly after isolation, the actual viable predator dose was known only a few days after each experiment, as the PFU appeared. Therefore, in some experiments, mainly involving *M. aeruginosavorus*, the inoculation sizes differed somewhat. The actual predator inoculation doses are specified for each experiment.

### **Rats**.

Wild-type male Sprague-Dawley (SD) rats (4 to 6 weeks old) were purchased from Charles River Laboratories (Wilmington, MA). All rats were housed under pathogen-free conditions at the Rutgers New Jersey Medical School animal facility. Guidelines from the Rutgers New Jersey Medical School Institutional Animal Care and Use Committee (protocol 15012) and the Animal Care and Use Review Office of the U.S. Army Medical Research and Material Command were followed in handling the animals.

### **Intranasal inoculation of bacteria**.

Predatory bacteria were introduced into the lungs of SD rats by intranasal inoculation to model a respiratory infection. After animals were anesthetized with 4% isoflurane–oxygen for 5 min using an isoflurane vaporizer, 50 µl of purified bacterial suspension was applied at both nostrils using a pipette. Rats were inoculated with PBS, *B. bacteriovorus* strain 109J, *B. bacteriovorus* strain HD100, *M. aeruginosavorus* strain ARL-13, or *K. pneumoniae*. To avoid cross contamination, animals were caged according to treatment group and time point to be sacrificed. After every set of inoculations was performed in each experiment, animals were visually assessed for signs of infection, illness, and discomfort. At 1, 24, and 48 h and 10 days postinoculation, lung, liver, spleen, and kidney samples were harvested for histological examination, inflammatory protein analysis, and bacterial dissemination experiments.

### Histological examination.

Lung samples designated for histology were stored in formalin at 4°C before examination. All histopathological examinations were performed by a pathologist blind to each specimen’s treatment group. Formalin-fixed organ segments from infected mice were paraffin embedded and stained with hematoxylin and eosin (H&E) for analysis of cellular composition as previously described. Stained sections were analyzed and photographed using an EVOS FL cell imaging system (Life Technologies, Carlsbad, CA).

### Inflammatory protein analysis (ELISA).

Lung samples were harvested in Lysing Matrix D tubes (MP Biomedicals) containing 1.0 ml of PBS with protease inhibitor. Samples were homogenized at 5.0 m/s for 30 s on a FastPrep-24 instrument (MP Biomedicals) before being stored at −80°C. At the time of analysis, homogenized tissues were thawed and centrifuged at >13,000 × *g* relative centrifugal force (RCF) for 10 min at 4°C. The resulting supernatant was filtered through a 0.22 µm-pore-size filter at 12 × *g* RCF for 4 min. Inflammatory proteins were measured using a V-Plex proinflammatory Panel 2 (rat) kit (K15059D-1; Meso Scale Discovery) according to the manufacturer’s instructions and read on a Sector Imager 2400 instrument (Meso Scale Discovery).

### Nucleic acid extraction.

Samples were prepared as previously described (37). Lung, liver, spleen, and kidney samples designated for nucleic acid extraction were harvested in Lysing Matrix D tubes containing 1.0 ml of TRIzol (Invitrogen). Samples were homogenized at 5.0 m/s for 30 s on a FastPrep-24 instrument before being stored at −80°C. Total RNA was extracted as previously described. Briefly, liquefied samples were spun down at >13,000 × *g* RCF for 20 min at 4°C to remove tissue debris. Two hundred microliters of chloroform was added to the supernatants, and the reaction mixture was centrifuged again at >13,000 × *g* RCF for 15 min at 4°C. An equal volume of isopropanol was added to the aqueous phase, and then the reaction mixture was centrifuged at >13,000 × *g* RCF for 15 min to pellet the precipitated RNA. After removal of the remaining isopropanol, pellets were washed twice with 500 ml of ice-cold 70% ethanol and then resuspended in 30 µl of nuclease-free water. Total RNA was then purified using the “RNA Cleanup” protocol described in the instructions provided for the RNeasy minikit (Qiagen) and stored at −80°C.

### Bacterial dissemination.

As organs were originally stored in TRIzol for gene expression analysis studies to be performed in the future, only RNA was available for dissemination analysis. cDNA synthesis was performed on total RNA isolated using iScript Reverse Transcription Supermix (Bio-Rad Laboratories) according to manufacturer’s instructions. The following primers specifically targeting the 16S rRNA gene of each predatory bacterial strain were synthesized: for *B. bacteriovorus* 109J and HD100 (38), (Forward) 5′-GGAGGCAGCAGTAGGGAATA-3′ and (Reverse) 5′-GCTAGGATCCCTCGTCTTACC-3′; for *M. aeruginosavorus* ARL-13, (Forward) 5′-GGCTTCACTTTGTCCAGAGC-3′ and (Reverse) 5′ CAGAAAAACGCGAAATCCTC 3′; for *K. pneumoniae* (39), (Forward) 5′ AGCACAGAGAGCTTGC 3′ and (Reverse) 5′ ACTTTGGTCTTGCGAC 3′. qPCR was performed on the samples in triplicate, with each reaction mixture containing the following components: template (1.0 µl of cDNA synthesized as described above), SsoAdvanced Universal SYBR green Supermix (Bio-Rad Laboratories), and a 500 nM (for 109J and *Micavibrio*) or 900 nM (for HD100) concentration of each primer (synthesized at the Rutgers New Jersey Medical School Molecular Resource Facility). A CFX384 Touch real-time PCR detection system (Bio-Rad Laboratories) was used with the following protocol: 50°C for 2 min (1 cycle), 95°C for 10 min (1 cycle), 95°C for 15 s and 60°C for 1 min (40 cycles), and 95°C for 15 s, 60°C for 15 s, and 95°C for 15 s (1 cycle). For each qPCR run, a 10-fold dilution series of the standard (purified DNA from each predatory strain) was assessed in triplicate to validate qPCR performance and facilitate quantification (E = 97%, *R*^2^ = 0.970, slope = −3.397). In addition, each qPCR run included negative controls (no template). 16S rRNA copy numbers were calculated using “Calculator for determining the number of copies of a template” (URI Genomics and Sequencing Center; http://cels.uri.edu/gsc/cndna.html) (40).

### Pathogen inoculation and treatment.

Animals were anesthetized and inoculated intranasally with 50 µl of *K. pneumoniae* as previously described. A 50-µl volume of predatory bacteria was administered to rats at 30 min and 6, 12, and 18 h postinfection for a total of four doses over a 24-h period. At 24 h postinoculation, animals were euthanized and lung samples harvested for histological examination and CFU plating. Lung samples designated for CFU were stored in Lysing Matrix D tubes containing 1.0 ml of PBS and placed on ice. Samples were immediately homogenized at 6.0 m/s for 1 min on a FastPrep-24 instrument. Liquefied samples were then serially diluted and plated on MacConkey agar in order to determine *K. pneumoniae* concentrations. Histological examination was performed as described above.

### Statistical analysis.

ELISA data are presented as means ± standard errors of the means; significant differences between the data from the treated samples and the data from the respective controls were determined using analysis of variance (ANOVA). *K. pneumoniae* reduction data are presented as medians; significant differences between treatment groups and respective controls were analyzed using the Mann-Whitney test. A *P* value of <0.05 was considered significant. Graphs were prepared and statistical analyses were performed using GraphPad Prism 6.05.
